# Lens dislocation delaying the diagnosis of metastasis to the optic nerve

**DOI:** 10.11604/pamj.2013.16.66.2165

**Published:** 2013-10-23

**Authors:** Cherkaoui Mandour, Mohammed Boucetta

**Affiliations:** 1 Departement Of Neurosurgery, Military Hospital Mohammed V, Rabat, PC: 10000 Morocco

**Keywords:** Metastasis, eye, optic nerve, lens dislocation

## Image in medicine

Metastasis to the eye is rare, it can affect almost all ocular tissues. The association of metastasis to the optic nerve and lens dislocation in the same orbit is exceptional. We repport a 63 years old man presented with blindness due to a lens dislocation in the left eye. One month later, he presented a right hemiparesis. Brain magnetic resonance imaging (MRI) showing brain and intra orbital metastasis associated with a lens dislocation. The histological result was provided by a stereotactic biopsy. The eye may be the site of several lesions to both. A lens dislocation can retarded a diagnosis of the nerve optic metastasis..

**Figure 1:**
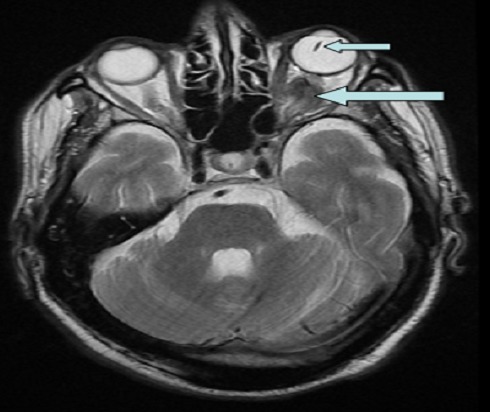
Brain magnetic resonance imaging (MRI) showing metastasis of the optic nerve associated with a lens dislocation

